# Risk Perception, Unhealthy Behavior, and Anxiety Due to Viral Epidemic Among Healthcare Workers: The Relationships With Depressive and Insomnia Symptoms During COVID-19

**DOI:** 10.3389/fpsyt.2021.615387

**Published:** 2021-03-19

**Authors:** Jukab Lee, Hyuk Joo Lee, Youjin Hong, Yong-Wook Shin, Seockhoon Chung, Jangho Park

**Affiliations:** ^1^Department of Psychiatry, Ulsan University Hospital, University of Ulsan College of Medicine, Ulsan, South Korea; ^2^Department of Psychiatry, GangNeung Asan Hospital, University of Ulsan College of Medicine, Gangneung, South Korea; ^3^Department of Psychiatry, Asan Medical Center, University of Ulsan College of Medicine, Seoul, South Korea

**Keywords:** COVID-19, healthcare worker, mental health problem, occupational stress, SAVE-9, online survey, hazardous work unit

## Abstract

We aimed to investigate the relationship between mental health problems and unhealthy behaviors among healthcare workers in response to the COVID-19 pandemic. Using an online survey, we collected data on healthcare workers' perception regarding COVID-19 exposure in a work unit. Workers' depression, insomnia, and anxiety symptoms were assessed using the Patient Health Questionnaire-9, Insomnia Severity Index, and Generalized Anxiety Disorder-7 scale, respectively. Work-related stress and anxiety in response to the viral epidemic were measured using the Stress and Anxiety to Viral Epidemic-9 (SAVE-9) scale. We found that work-related stress and anxiety in response to the viral epidemic was associated with female sex, perception of the workplace as being dangerous, and depressive symptoms. Unhealthy behaviors, such as smoking and drinking as coping behaviors during the pandemic, were associated with male sex, young age, depression, and insomnia. During the COVID-19 pandemic, it is necessary to closely observe the patterns of work-related stress and anxiety reactions among healthcare workers to reduce their burnout.

## Introduction

The coronavirus disease (COVID-19) pandemic, which began in 2019 (1), is still spreading to date. As of October 5, 2020, the cumulative number of confirmed cases worldwide was approximately 35 million, with ~1 million fatalities (2). Health authorities and citizens have been implementing quarantine measures, but the spread of COVID-19 has not been contained; the number of confirmed cases and deaths continues to increase. Despite the low mortality rate of 2%, the severe acute respiratory syndrome coronavirus 2 has a high transmission rate; therefore, it has a higher mortality rate than those of severe acute respiratory syndrome (SARS) and Middle East respiratory syndrome (MERS), combined (3). To end this pandemic, a treatment measure or vaccine is needed. Many countries have been attempting to develop vaccines, but without much success (4, 5). On August 10, 2020, Russia announced that they had developed the first COVID-19 vaccine; however, its effectiveness remains unclear (6). Therefore, it is difficult to predict the end of the COVID-19 pandemic, and it seems likely to last for a long time.

In Korea, the first confirmed case of COVID-19 was reported in January 2020. Since February, the number of cases has increased exponentially due to infections traced to a religious organization in Daegu, Korea (7). However, through a strong social distancing policy and a “test-trace-isolate” strategy, the number of confirmed cases was controlled relatively well, and this appeared to be a more successful strategy in implementing COVID-19 quarantine compared to other countries (8). Nevertheless, several experts anticipated a second outbreak after autumn, and sooner than expected (9), after a large-scale urban rally in mid-August, there was an increase in the confirmed cases nationwide. The efforts and dedication of healthcare workers (HCW) have been essential to the successful COVID-19 quarantine in South Korea; however, these HCW may be prone to mental health problems in these difficult situations.

A pandemic of infectious diseases can cause a variety of serious psychological symptoms (10). In the general population, during the pandemic period, existing psychiatric symptoms may worsen, or new ones may appear. Furthermore, excessive worry, anxiety, helplessness, and a tendency to blame the infected may also occur. Some people also experience depression, anxiety, panic attacks, physical symptoms, and suicidal thoughts (11–14). As the COVID-19 pandemic continues to spread, “Corona Blue,” an experience of depression due to a decrease in social activities, isolation, and economic downturn, has already begun among the public (15). According to a survey, 4 out of 10 adults suffer from Corona Blue in Korea (16). As such, psychological difficulties among HCW treating COVID-19 patients in hospitals are even greater.

HCW play several important roles in hospitals, such as responding to suspected or confirmed COVID-19 patients, testing, and treatment. In addition to intensive labor, they face many difficulties, including a shortage of alternative workers, exhaustion due to wearing protective equipment for a long time, a high risk of infection, and prejudice surrounding them as potential sources of infection (17). If the COVID-19 pandemic is prolonged, HCW who must work face-to-face with confirmed or suspected COVID-19 patients could experience mental health issues such as burnout, depression, and anxiety, as well as adjustment issues due to changes in work or increased work load (18–20).

Since the COVID-19 pandemic, there have been several studies on HCW, and many have indicated that they are at a higher risk of infection than the general population (21); therefore, they should be protected. There have also been several studies on HCW's mental health issues caused due to COVID-19. For instance, in a study by Kang et al., of the 994 medical and nursing staff studied, 36.9% had subthreshold, 34.4% had mild, 22.4% had moderate, and 6.2% had severe mental health disturbances (12). Furthermore, a study by Lai et al. found that 50.4% of the 1,257 HCW reported depressive symptoms, 44.6% reported anxiety, 34.0% reported insomnia, and 71.5% reported distress (22). Alternatively, in a study by Xiao et al., it was found that the higher the social support levels of HCW, such as care or support from other people; self-efficacy; and sleep quality, the lower their anxiety and stress levels (23). Ahn et al. further reported that depression, general anxiety, and virus-related anxiety among nurses working in hospitals treating COVID-19 were significantly higher than those in other HCW; the risk factors for depression included nurse occupation, being single, having higher levels of anxiety, and experiencing stress related to the viral infection (24).

In this study, we hypothesized that HCW would face mental health issues in the COVID-19 pandemic situation, and that these would be related to how hazardous they perceive their workplaces to be and their unhealthy behavior. Thus, we investigated the relationship between mental health problems among HCW due to COVID-19 and occupation, socio-demographics, workplace, degree of contact with COVID-19 patients, and unhealthy behavior.

## Methods

### Participants and Procedure

This study was conducted in Ulsan University Hospital located in Ulsan metropolitan city, Korea. Ulsan University Hospital has more than 1,000 beds, conducting COVID-19 screening tests 24 h a day, and is in charge of treating COVID-19 patients in Ulsan metropolitan city, which has a population of 1.13 million. We developed an online survey for assessing HCW's mental health, work-related stress, and coping behavior during the pandemic. We advertised this study via the hospital's intranet, and enrolled HCW who were working in the hospital from June 22 to July 8, 2020. The protocol of this study was approved by the Institutional Review Board of Ulsan University Hospital (UUH 2020-06-021), and written informed consent for participation in this online survey was waived.

Via the online survey, we collected information on participants' age, sex, marital status, occupation, and years of employment. We also asked participants the following questions: “Do you have experience of taking care of the COVID-19 confirmed patients?”, “Did you experience being quarantined due to infection with COVID-19?”, and “Are you experiencing or have experienced treated depression, anxiety, or insomnia?”

### Assessment

#### Risk Perception of COVID-19 Exposure in a Work Unit

To assess workers' consideration of their workplace with the risk associated with COVID-19 exposure, we asked participants the following question: “Do you think that your work unit is at higher risk of infection than others?” The answers were on a five-point scale with the following options: 1 - strongly disagree, 2 - disagree, 3 - neutral, 4 - agree, 5 - strongly agree.

#### Patient Health Questionnaire-9 Items (PHQ-9)

The Patient Health Questionnaire-9 (PHQ-9) is a self-rating scale for assessing depressive symptoms (25). It consists of 9 items scored on a Likert scale (0 = not at all to 3 = nearly every day). Total scores can range from 0 to 27, with higher scores reflecting greater symptom severity (0–4 = minimal depression, 5–9 = mild depression, 10–14 = moderate depression, 15–19 = moderately severe depression, and ≥20 = severe depression). A PHQ-9 score ≥10 was reported to have a sensitivity and specificity of 88% for major depression (26). In this study, we defined major depression as having a PHQ-9 score ≥10.

#### Generalized Anxiety Disorder-7 Items (GAD-7)

The Generalized Anxiety Disorder-7 (GAD-7) is a self-rating scale for assessing general anxiety symptoms (27). It consists of 7 items scored on a Likert scale (0 = not at all to 3 = nearly every day). Total scores can range from 0 to 21, with higher scores reflecting higher levels of anxiety. Cut-off intervals for anxiety include 0–4 (minimal), 5–9 (mild anxiety), 10–14 (moderate), and 15–21 (severe) (28). For the purposes of the current study, we used a cut-off of five or higher to identify cases associated with anxiety.

#### Stress and Anxiety to Viral Epidemic - 9 (SAVE-9) Scale

The Stress and Anxiety to Viral Epidemic - 9 (SAVE-9) is a new scale for assessing anxiety and stress among HCW in response to the viral pandemic. It consists of two subcategories: (1) anxiety about the viral pandemic (SAVE-6) and (2) work-related stress associated with the viral pandemic. Respondents rated their agreement with each item on a five-point Likert scale with scores from 0 (never), 1 (rarely), 2 (sometimes), 3 (often), and 4 (always) (29). The cut-off points for the SAVE-9 and SAVE-6 scales for mild degrees of GAD-7 (score ≥ 5) were observed to be 21 and 15, respectively (30).

#### Insomnia Severity Index (ISI)

The Insomnia Severity Index (ISI) consists of seven items for assessing the severity of insomnia (31). A five-point Likert scale is used to rate each item (0 = no problem, 4 = very severe problem), yielding a total score ranging from 0 to 28. The total score is interpreted as follows: absence of insomnia (0–7), sub-threshold insomnia (8–14), moderate insomnia (15–21), and severe insomnia (22–28). Validation of the ISI as an outcome measure for insomnia research (32), and the cut-off for insomnia using this scale is a score of eight.

### Statistical Analysis

Statistical analyses were conducted with SPSS ver. 21.0 for Windows (IBM Corp., Armonk, NY). The clinical characteristics are summarized as the mean ± standard deviation. The level of significance for all analyses was defined as two-tailed *p* < 0.05. Student's *t*-test for continuous variables and Chi-square test for categorical variables were conducted for between groups analyses. Spearman correlation analysis was conducted for the relationship between clinical characteristics and mental health symptoms, since the PHQ-9 and GAD-7 scales were not within the normal distribution confirmed by Kolmogorov-Smirnov test. Logistic regression analysis was performed to explore the expected factors for depression, stress, and anxiety in response to the viral pandemic, as well as determine unhealthy behaviors among HCW.

## Results

All 406 (15.1%) of 2,683 HCWs working at Ulsan University Hospital voluntarily responded to the online survey. As can be seen in Table 1, among the respondents, 226 (55.7%) were nursing professionals, 291 (71.7%) female, and 255 (62.8%) married, and the mean year of employment was 11.0 ± 9.2 years. Seventy-four (18.2%) workers had experience in taking care of confirmed COVID-19 patients, 27 (6.7%) experienced being quarantined due to being infected with COVID-19, and 27 (6.7%) had or had experienced treated depression, anxiety, or insomnia. Of the 406, 109 (26.8%) responded with 'agree' and 35(8.6%) with 'strongly agree' in risk perception. The mean score of the PHQ-9 was 4.9 ± 4.3, GAD-7 was 4.0 ± 3.5, and SAVE-9 was 20.1 ± 5.5. Among HCW, 58 (14.3%) were rated as having depression (PHQ-9 score ≥ 10), 160 (39.4%) as having mild degrees of general anxiety (GAD-7 ≥ 5), 211 (52.0%) as having anxiety responses to the viral pandemic (SAVE-9 ≥ 21), and 147 (36.2%) as having insomnia (ISI ≥ 8). When about their coping behaviors in response to the stress experienced during the COVID-19 pandemic, the workers identified the following behaviors: having conversations with people (*n* = 252, 62.1%), partaking in hobbies and exercise (*n* = 225, 55.4%), partaking in unhealthy behaviors such as smoking and drinking (*n* = 74, 18.2%), and using social network services (SNS) via internet (*n* = 31, 7.6%).

**Table 1:** Demographic characteristics of subjects (*N* = 406).

**Healthcare workers**	
Medical doctors	81 (20.0%)
Nursing professionals	226 (55.7%)
Other healthcare workers	99 (24.3%)
Sex (female)	291 (71.7%)
**Age**	
20–29	93 (22.9%)
30–39	132 (32.5%)
40–49	126 (31.0%)
50–65	55 (13.5%)
**Marital status**	
Single	151 (37.2%)
Married without kids	33 (8.1%)
Married with kids	222 (54.7%)
Do you have experience in taking care of confirmed COVID-19 patients? (Yes)	74 (18.2%)
Did you experience being quarantined due to infection with COVID-19? (Yes)	27 (6.7%)
Do you think that your workplace is at higher risk of infection than other workers' units?	3.0 ± 1.1
Do you currently have or had treated depression, anxiety, or insomnia? (Yes)	27 (6.7%)
Years of employment (year)	11.0 ± 9.2
**Coping behavior to the stress**	
Having conversation with people	252 (62.1%)
Unhealthy behavior such as smoking and drinking	74 (18.2%)
Hobby & exercise	225 (55.4%)
Using the Social network services (SNS) via internet	31 (7.6%)

### Risk Perception and Anxiety Response to the Viral Epidemic

When we considered the significance of the *p*-value as 0.0167 for multiple comparisons, nursing professionals felt their work unit was more risky, female HCW and junior HCW suffered more from stress and anxiety to the viral epidemic, and workers who had experience treating confirmed COVID-19 patients or who had experienced being quarantined were more afraid that their workplace was a risk for being infected by COVID-19 than other workers (refer to Table 2 for a detailed summary of the results).

**Table 2:** Healthcare workers' risk perception and anxiety response to the viral epidemic (*N* = 406).

**Variables**	**Risk perception of HCWs**		**Work-related stress** **subcategory of SAVE-9**	
	**Mean ± SD**	***P*-value**	**Mean ± SD**	***P*-value**
**Healthcare workers**				
Nursing professionals (*N* = 226)	3.2 ± 1.1	0.005	20.8 ± 5.3	0.003
Other healthcare workers (*N* = 180)	2.9 ± 1.0		19.2 ± 5.6	
**Sex**				
Male (*N* = 115)	3.0 ± 1.1	0.43	18.0 ± 5.6	<0.001
Female (*N* = 291)	3.1 ± 1.1		21.0 ± 5.2	
**Age**				
20–39 (*N* = 225)	3.1 ± 1.1	0.048	20.8 ± 5.4	0.004
40–65 (*N* = 181)	2.9 ± 1.0	0	19.3 ± 5.5	
**Marital status**				
Single (*N* = 151)	3.2 ± 1.1	0.20	20.4 ± 5.2	0.69
Married without kids (*N* = 33)	2.9 ± 1.0		19.9 ± 5.3	33 (8.1%)
Married with kids (*N* = 222)	3.0 ± 1.0		20.0 ± 5.5	222 (54.7%)
**Did you have experience taking care of confirmed COVID-19 patients?**				
Yes (*N* = 74)	3.7 ± 1.1	<0.001	20.8 ± 5.6	0.25
No (*N* = 332)	2.9 ± 1.0		20.0 ± 5.4	27 (6.7%)
**Did you experience being quarantined due to infection with COVID-19?**				
Yes (*N* = 27)	3.7 ± 1.1	0.001	19.5 ± 5.9	0.53
No (*N* = 379)	3.0 ± 1.0		20.2 ± 5.4	
**Unhealthy behavior such as smoking and drinking**				
Yes (*N* = 74)	3.3 ± 1.0	0.047	21.5 ± 5.2	0.016
No (*N* = 332)	3.0 ± 1.1		19.8 ± 5.5	

In the Spearman correlation analysis, the degree of considering a workplace as hazardous and of having high levels of stress and anxiety due to the viral epidemic were significantly correlated with being younger and having higher levels of depression, anxiety, and insomnia. Furthermore, anxiety due to the viral epidemic and the degree of considering a workplace as hazardous were correlated with each other (refer to Table 3 for the correlations).

**Table 3:** Spearman's correlation coefficients of each variables.

**Variables**	**Age**	**Year of employment**	**PHQ-9**	**SAVE-9**	**GAD-7**	**ISI**	**Risk perception**
Age	1.000						
Year of employment	0.75^**^	1.000					
PHQ-9	−0.25^**^	−0.19^**^	1.000				
SAVE-9	−0.14^**^	−0.03	0.34^**^	1.000			
GAD-7	−0.25^**^	−0.22^**^	0.69^**^	0.39^**^	1.000		
ISI	−0.23^**^	−0.21^**^	0.62^**^	0.33^**^	0.52^**^	1.000	
Risk perception	−0.11^*^	−0.03	0.13^**^	0.32^**^	0.22^**^	0.12^*^	1.000

*PHQ-9, Patient Health Questionnaire-9; SAVE-9, Stress and Anxiety to Viral Epidemic-9 items; GAD-7, Generalized Anxiety Scale-7 items; ISI, Insomnia Severity Index*.

* *p < 0.05*,

** *p < 0.01*.

### Risk Perception, Anxiety Due to Viral Epidemic, and Unhealthy Behaviors

As can be seen in Table 4, HCWs' risk perception was elevated by experienced taking care of confirmed patients [aOR = 3.72, 95% CI (2.13–6.50)], being quarantined [aOR = 2.60, 95% CI (1.06–6.39)], and high stress and anxiety due to the viral epidemic [aOR = 1.12, 95% CI (1.07–1.17)]. Their stress and anxiety in response to the viral pandemic (SAVE-9 score) was related to sex-female [aOR = 2.36, 95% CI (1.47–3.80)], risky perception score [aOR = 1.50, 95% CI (1.21–1.84)], and experiencing or having experienced depression [aOR = 1.14, 95% CI (1.08–1.21)]. HCW unhealthy behaviors, such as smoking and drinking, were related to sex-male [aOR = 2.11, 95% CI (1.17–3.80)], younger age (20–39 years old) [aOR = 1.92, 95% CI (1.06–3.46)], experiencing or having experienced depression [aOR = 1.09. 95% CI (1.01–1.17)], and insomnia [aOR = 1.07, 95% CI (1.01–1.14)].

**Table 4:** Logistic regression analysis to explore expecting variables for workers' mental health.

	**Crude OR**	**95% CI**	**Adjusted OR**	**95% CI**
Explanatory variables: Risk perception (agree and strongly agree)^*^				
Experienced taking care of confirmed patients (yes)	3.92	2.31–6.64	3.72	2.13–6.50
Experienced being quarantined (yes)	2.85	1.29–6.33	2.60	1.06–6.39
Stress and Anxiety to Viral Epidemic-9 items (SAVE-9) score	1.17	1.09–1.16	1.12	1.07–1.17
Explanatory variables: SAVE-9 ≥ 21^**^				
Sex (female)	2.54	1.62–4.00	2.36	1.47–3.80
Risk perception score	1.53	1.26–1.86	1.50	1.21–1.84
Patient Health Questionnaire-9 items (PHQ-9) score	1.16	1.10–1.22	1.10	1.03–1.18
Explanatory variables: Unhealthy behavior^***^				
Sex (male)	1.48	1.01–2.53	2.11	1.17–3.80
Junior (20~39 years old)	2.35	1.36–4.07	1.92	1.06–3.46
Patient Health Questionnaire-9 items score	1.15	1.09–1.21	1.09	1.01–1.17
Insomnia Severity Index score	1.12	1.07–1.18	1.07	1.01–1.14

* *Logistic regression analysis with forward (conditional) method was performed, and age, marital status, job, years of employment, unhealthy behavior, patient health questionnaire-9 items score, and insomnia severity index score were excluded in the final model*.

** *Logistic regression analysis with forward (conditional) methods was performed, and age, marital status, job, years of employment, unhealthy behavior, and insomnia severity index score were excluded in the final model*.

*** *Logistic regression analysis with forward (conditional) method was done, and marital status, job, years of employment, risky perception score, and stress and anxiety due to viral epidemic scale score were excluded in the final model*.

## Discussion

This study investigated mental health problems experienced by HCW in hospitals treating suspected or confirmed COVID-19 patients. Results indicated that a significant number of HCW experienced mental health issues such as depression, anxiety, and insomnia, consistent with recently published studies. We analyzed these mental health issues according to occupation, socio-demographic information, workplace, contact with COVID-19 patients, and unhealthy behaviors. It was found that sex, occupational group, length of service, contact with confirmed patients, and perception of the workplace being hazardous were related to mental health problems.

During the pandemic, HCW who were at the frontline suffered from mental health problems such as stress, anxiety, and depression (33). In this study, 58 (14.3%) workers were rated as having depression (PHQ-9 score ≥ 10), 160 (39.4%) had mild degrees of general anxiety (GAD-7 ≥ 5), 211 (52.0%) had anxiety responses to the viral epidemic (SAVE-9 ≥ 21), and 147 (36.2%) experienced insomnia (ISI ≥ 8). Our findings are consistent with the study of Lai et al. (22) which found that 50.4% of the study participants reported depressive symptoms, 44.6% reported anxiety, 34.0% reported insomnia, and 71.5% reported distress. This was also consistent with the research by Kang et al. (34), where most of the medical and nursing staff working at Wuhan complained of mental health disturbances to varying degrees ranging from subthreshold to severe.

In this study, being female was a significant predictor for HCW work-related stress and anxiety in response to COVID-19. This is understandable because women generally have higher anxiety reactions than men (35); thus, their work-related stress and anxiety related to COVID-19 are likely to be greater (24). Although it was not statistically significant, it was found that nursing professionals suffered more work-related stress compared to other HCW in this study. In our previous study (24), we also observed that nursing professionals showed significantly higher levels of depression and anxiety, and had higher SAVE-9 scale scores than other HCW groups. Nursing professionals care for and spend the most time with COVID-19-infected patients treated in the hospital; due to this, they fear becoming infected themselves. In addition, nursing professionals who care for infected patients must wear protective equipment. Putting on and taking off this equipment is not only very difficult, but is also time-consuming, which takes time away from their time to rest. Wearing heavy protective equipment throughout the day is physically strenuous. Even when dealing with suspected patients who are not infected, they tend to feel anxious because of close contact. Furthermore, there is a possibility that nurses' anxiety about infection is higher because their knowledge about infectious diseases is insufficient compared to that of medical doctors. For this reason, nursing professionals seem to have the highest work-related stress.

HCW who came in direct contact with infected patients showed higher levels of depression than those who did not; the higher their levels of depression, the higher the levels of work-related stress and anxiety in response to COVID-19. In general, depression and anxiety are closely related, so it is understandable that anxiety is easily experienced when depression is high (36). Considering that depression is also associated with insomnia, the mental health of the group facing infected patients was more at risk. HCW who had experience in treating or quarantining infected patients, recognized their workplace as having a higher risk of infection, and considered their workplace more hazardous. These experiences and subjective risk perception regarding infectious hazard were significant predictors for HCW work-related stress and anxiety in response to COVID-19. The tendency to perceive one's workplace as a more dangerous place may be due to working in a more risky environment, while increased anxiety and subjective perception of infectious hazard may be due to the experience of facing COVID-19 patients or being quarantined. Taken together, HCW who worked with infected patients were more depressed, perceived their workplace as being more dangerous, and experienced more anxiety and work-related stress.

The younger HCW (aged 20–39) experienced more work-related stress, but the longer they worked, the less they experienced depression, anxiety, and insomnia. Considering that age is related to working period, being younger and having shorter working periods makes individuals more susceptible to mental health problems such as depression, anxiety, insomnia, and having higher levels of work-related stress. Therefore, HCW who have gained a lot of experience through long-term work, seem to respond relatively well to the problems encountered at work.

During the COVID-19 pandemic, unhealthy behaviors such as drinking alcohol and smoking were associated with being male, younger age (aged 29–30), depression, and insomnia. Our study found that women felt more anxious and had work-related stress caused by the COVID-19 pandemic; however, men easily displayed unhealthy behaviors. This is because men are generally more likely to try to relieve stress by engaging in unhealthy behaviors, such as drinking alcohol and smoking, compared to women (37); it is posited that the younger a person is, the less likely they are to engage in unhealthy behaviors due to a lack of response strategies or support systems in stressful situations. For instance, when men experience mental health problems such as depression and insomnia and do not receive appropriate treatment, they tend drink or smoke. These unhealthy behaviors can worsen symptoms such as depression and insomnia. In general, not during a pandemic, unhealthy behaviors such as drinking and smoking are related to sex-men, depression, and insomnia, which is consistent with our results.

In our study, women and nursing professionals were more vulnerable to stress and depression during the pandemic. It has been suggested that a mental health and stress management program is needed to assist nurses in caring for infected patients (38, 39). Training on infectious diseases, infection prevention, and wearing protective equipment will help reduce the work-related stress (40). Furthermore, HCW who are relatively young and have shorter working periods are more vulnerable to mental health and have more stress. In a pandemic, it appears that these HCW need more resources for support and training effectively. Since HCW who interact with confirmed COVID-19 patients in person consider their workplace to be riskier, are more anxious, and have more work-related stress, it is necessary to adopt measures to mitigate these effects for HCW as they will likely continue to face COVID-19 patients in hospitals. Furthermore, as mentioned before, young, male HCW tend to relieve stress by engaging in unhealthy behavior; therefore, it is necessary to develop a healthier stress relief strategy or support system for these workers.

This study has several strengths. The sample size was large and a variety of HCW were included. Multiple comparison was considered in the analyses. This was one of the rare studies on this issue in the COVID-19 pandemic and provides some fundamental information regarding healthcare workers' mental health during the pandemic, which may help design guidelines or strategies to cope with such issues in the midst of this type of specialized situation. Additionally, research safety was guaranteed by appropriately utilizing an online survey.

The limitations of this study are as follows. First, this is a single-center study. Second, the general population as a control group did not participate in this study, although we can easily expect that anxiety and stress levels of HCW are higher than the general population. Third, shift workers were also included among the HCWs who responded to our study's online survey. Although shift work may affect mental health problems such as insomnia, this was not statistically corrected. Fourth, as a limitation of conducting a self-report survey study, a fully detailed interview was not done. However, during this pandemic, an online survey rather than face-to-face interviews was necessary to prevent the spread of the virus. Fifth, we could not ask and gather objective data, such as number of patients and the characteristics of the workplace, when we assessed the risk of the work unit. Tt is not easy to gather the data via on-line survey, and we wanted to measure the subjective risk perception on the HCWs work units rather than the real risk.

In conclusion, female and junior HCW suffer more from work-related stress. Work-related stress and anxiety in response to the pandemic was related to being female, percieving the workplace as dangerous, and experiencing depression. Unhealthy behaviors such as smoking and drinking as coping behaviors during the pandemic were related to being male, young, experiencing depression, and having insomnia. Thus, during the COVID-19 pandemic, it is necessary to closely observe the patterns of work-related stress and anxiety reactions among HCW, and devise a plan to reduce their burnout.

## Data Availability Statement

The raw data supporting the conclusions of this article will be made available by the authors, without undue reservation.

## Author Contributions

JL drafted or provided critical revision of the article. HL performed experiments and analysis. YH provided final approval of the version to publish. Y-WS agreed to be accountable for all aspects of the work in ensuring that questions related to the accuracy or integrity of any part of the work are appropriately investigated and resolved. SC and JP contributed substantially to the conception and design of the study, the acquisition of data, and or the analysis and interpretation. All authors contributed to the article and approved the submitted version.

## Conflict of Interest

The authors declare that the research was conducted in the absence of any commercial or financial relationships that could be construed as a potential conflict of interest.
